# Secondary Metabolites Produced by an Endophytic Fungus *Pestalotiopsis sydowiana* and Their 20S Proteasome Inhibitory Activities

**DOI:** 10.3390/molecules21070944

**Published:** 2016-07-20

**Authors:** Xuekui Xia, Soonok Kim, Changheng Liu, Sang Hee Shim

**Affiliations:** 1 Key Biosensor Laboratory of Shandong Province, Biology Institute, Shandong Academy of Sciences, Jinan 250014, China; summerxkgao@163.com (X.X.); changhengliu@163.com (C.L.); 2 National Institute of Biological Resources, Incheon 22689, Korea; sokim90@gmail.com; 3 Duksung IDC, College of Pharmacy, Duksung Women’s University, Seoul 01369, Korea

**Keywords:** *Pestalotiopsis sydowiana*, secondary metabolites, 20S proteasome inhibitory activities

## Abstract

Fungal endophytes have attracted attention due to their functional diversity. Secondary metabolites produced by *Pestalotiopsis sydowiana* from a halophyte, *Phragmites communis* Trinus, were investigated. Eleven compounds, including four penicillide derivatives (**1**–**4**) and seven α-pyrone analogues (**5**–**10**) were isolated from cultures of *P. sydowiana*. The compounds were identified based on spectroscopic data. The inhibitory activities against the 20S proteasome were evaluated. Compounds **1**–**3**, **5**, and **9**–**10** showed modest proteasome inhibition activities, while compound **8** showed strong activity with an IC_50_ of 1.2 ± 0.3 μM. This is the first study on the secondary metabolites produced by *P. sydowiana* and their proteasome inhibitory activities. The endophytic fungus *P. sydowiana* might be a good resource for proteasome inhibitors.

## 1. Introduction

The biological role of the ubiquitin proteasome pathway (UPP) is regarded to be very important in cancer therapy. The UPP is the major pathway for degradation of intracellular proteins, playing an important role in the regulation of cellular proteins that are critical in cell cycle regulation, signal transduction, gene transcription, and apoptosis [1,2]. Polyubiquitin is attached to a target protein, and the ubiquitinated protein is selected for degradation by proteasomes [3]. A eukaryotic 26S proteasome is composed of two subunits, a 20S core particle structure and two 19S regulator caps [4]. The number and diversity of subunits contained in the 20S core particle depend on the organism. All 20S particles consist of four stacked heptameric ring structures that are composed of two subunits, structural α subunits and catalytic β subunits [5]. The outer two rings in the stack consist of seven α subunits, and the inner two rings each consist of seven β subunits containing the protease active sites that perform the proteolysis reactions [5]. In bacterial and archaeal proteasomes, all α and β subunits are identical, whereas eukaryotic proteasomes contain seven distinct types of each subunit. In mammals, the β1, β2, and β5 subunits are catalytic and share a common mechanism. In eukaryotes, the 20S proteasome is both ubiquitous and essential. Some prokaryotes also share homologs of the 20S proteasome, whereas most bacteria possess heat shock genes, whose gene products are a multimeric protease and an ATPase [6]. In this study, we focused on mammalian proteasomes, especially human erythrocyte 20S. Proteasome inhibitors block the degradation of a significant number of regulated proteins, which induces intracellular signaling system disorders. Accumulation of ubiquitin has been observed in various cancers, indicating that a disturbance in the protein degradation process could influence tumor growth. In preclinical studies, cancer cells have a higher sensitivity to the pro-apoptotic effects of proteasome inhibition than do normal cells. Thus, proteasome inhibitors have potential for use as anticancer agents. Since approval of the first proteasome inhibitor, bortezomib (Velcade™, Millenium), by the US FDA for the treatment of multiple myeloma in 2003 [7], natural and synthetic products have been investigated as proteasome inhibitors and antitumor agents. Because some proteasome inhibitors, such as bortezomib, have undesirable side-effects, natural products with low toxicities have received attention for their potential in the discovery of anticancer agents as proteasome inhibitors. To date, three proteasome inhibitors, bortezomib, carfilzomib, and ixazomib, have been approved by the US FDA. Furthermore, some proteasome inhibitors, including marizomib (salinosporamide A, NPI-0052) [8] and CEP-18770, have entered clinical trials. Among them, carfilzomib and marizomib are derived from natural substances produced by microorganisms. Many natural products have been evaluated as potential proteasome inhibitors: withaferin A from *Acnistus arborescens*; celastrol from *Tripterygium wilfordii*; gliotoxin from several fungi, including *Aspergillus fumigatus*, *Trichoderma*, and *Penicillium*; and ginsenoside Rd from *Panax ginseng* [9]. Despite a number of studies on proteasome inhibitors, there is still a need to discover new types of proteasome inhibitors with suitable pharmacological properties.

As part of a project to discover natural proteasome inhibitors, several plant extracts were screened using a 20S proteasome inhibition assay. The endophytic strain *Pestalotiopsis sydowiana* was chosen for further chemical investigation because it exhibited potent 20S proteasome inhibitory activity in a preliminary study. Endophytes are microorganisms that spend all or part of their life cycle colonizing healthy tissues of their host [10]. Endophytes have attracted considerable attention due to their noticeable species and functional diversity [10]. Furthermore, they are known as potential sources of compounds with intriguing chemical structures and biological activities, some of which are potential candidates for drug development [11,12]. *Pestalotiopsis* is a ubiquitously distributed genus, occurring on a wide range of substrata. Interest in this fungus has increased considerably because it has produced many bioactive secondary metabolites to treat human diseases, as well as to control plant diseases [13]. The anticancer agent taxol has been discovered from this genus [14,15]. *P. sydowiana* was first found on azaleas and rhododendrons as a pathogen and was also isolated from the red bayberry (*Myrica rubra* Seib & Zucc.). In this study, this fungus was isolated from a halophyte, *Phragmites communis* Trinius, collected from a swamp area of Suncheon, South Korea. To the best of our knowledge, there are no published reports on secondary metabolites produced by the fungus *P. sydowiana*. In this study, commercially available human erythrocyte 20S proteasomes were used. The objective of this study was to isolate and identify secondary metabolites and evaluate their 20S proteasome inhibitory activities.

**Figure 1:**
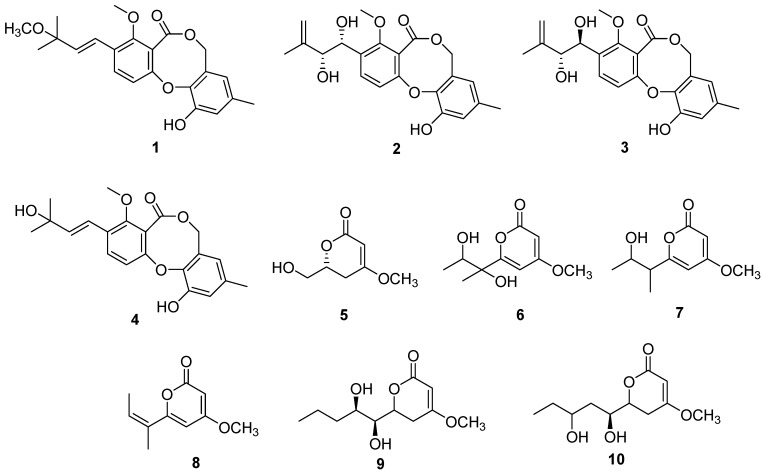
Structures of compounds **1**–**10**.

A solid culture of *P. sydowiana* on cooked rice was thoroughly extracted with ethyl acetate. The extracts were subjected to a series of column chromatographic methods, which led to the isolation of 10 polyketides (**1**–**10**) (Figure 1).

## 2. Results and Discussion

### 2.1. Structure Determination

Through a comparison of ^1^H-NMR, ^13^C-NMR, and ESIMS with the reported data, compounds **1**–**10** were identified as 3′-*O*-methyldehydroisopenicillide (**1**), pestalotiollide B (**2**), pestalotiollide A (**3**), dehydroisopenicillide (**4**) [16], 6-hydroxymethyl-4-methoxy-5,6-dihydro-2*H*-pyran-2-one (**5**) [17], pestalotiopyrone D (**6**), pestalotiopyrone E (**7**), pestalotiopyrone G (**8**) [18], LL-P880b (**9**) [19], and photipyrone B (**10**) [20]. Compound **5** was obtained from nature for the first time and had been synthesized as an intermediate just once before. Compounds **1**–**4** have the same skeleton as the penicillide derivatives bearing 7*H*-dibenzo[*b*,*g*]dioxocin-5-one. The hydroxyl group at 3′ of **4** was methylated in the side chain of **1,** and the hydroxyls joined at 1′ (δ_H_ 4.98, δ_C_ 69.1) and 2′ (δ_H_ 4.15, δ_C_ 78.2) in the same orientation. Protons at 1′ (δ_H_ 5.04, δ_C_ 69.2) and 2′ (δ_H_ 4.28, δ_C_ 78.7) were in different side chain orientations relative to compound **2**, while those at 1′ (δ_H_ 4.98, δ_C_ 69.1) and 2′ (δ_H_ 4.15, δ_C_ 78.2) were in the same orientation as in compound **3**.

Compounds **5**–**10** are α-pyrone analogues, and the skeleton could be elucidated by ^1^H- and ^13^C-NMR. Compounds **6**–**8** were previously isolated from *Pestalotiopsis* sp., and compounds **9**–**10** were previously reported from *Pestalotiopsis photiniae*. To our knowledge, this is the second report on the isolation of compounds **6**–**8** and **10** from nature. Compounds **5**–**10** have different side chains: hydroxymethylene in **5**; 1′,2′-dihydroxy-1′-methyl-propane in **6**; 2′-dihydroxy-1′-methyl-propane in **7**; 2-methyl-propylene in **8**; 1,2-dihydroxy-butyl in **9**; and 1,3-dihydroxy-butyl in **10**.

### 2.2. 20S Proteasome Inhibitory Activities

All of the isolated compounds (**1**–**10**) were evaluated for inhibitory activity against the 20S proteasome (Table 1). Commercially available purified human erythrocyte 20S proteasomes and the substrate Suc-LLVY-AMC were used to determine the chymotrypsin-like activity of the 20S proteasome. Epoxomicin (IC_50_ = 72 nm), a known proteasome inhibitor, was used as a positive control. Most of compounds, except for compounds **4**, **6**, and **7**, inhibited the activity of the 20S proteasome in a dose-dependent manner, with IC_50_ values ranging from 1.2 ± 0.3 μM to 30.5 ± 1.5 μM.

Among the isolated compounds, those with an α-pyrone (**5**–**10**) exhibited more potent inhibitory activities against 20S proteasomes than the penicillide derivatives (**1**–**4**). Furthermore, compound **8**, with a double bond in the side chain, demonstrated more potent inhibitory activities compared with other α-pyrone analogues. In addition, compounds **9**–**10**, with long side chains, displayed moderate activities, even though they do not possess the double bond in the side chain. Himeic acid A, a ubiquitin-activating enzyme inhibitor derived from a marine fungus, also has a pyrone skeleton with a double bond in its long side chain, even though it has γ-pyrone instead of α-pyrone [21]. Presumably, the double bond in the side chain and/or the chain length of the pyrones might play an important role in proteasome inhibition. Most natural proteasome inhibitors are reported to have a carbonyl group, which is sometimes a member of a lactone ring or amide group. At the active site, β subunit of the proteasome, the hydroxyl group of Thr-1 was reported to either nucleophilically attack the carbonyl groups of the inhibitors, forming a covalent adduct, or form a hydrogen bond with them in the case of non-covalent inhibitors [22]. Among the proteasome inhibitors, a lactone group was reported to be an important functional group, as in omuralide and belactosin C, which might explain the moderate inhibitory activities of the isolated compounds in this study. In an X-ray study, syringolin A, a lactam with an α,β-unsaturated carboxamide group, was found to interact with the proteasome such that the amino-terminal Thr1 of the proteasome performed a Michael-type 1,4-addition to the double bond, resulting in a covalent ether bond [22]. The Michael addition was presumed to occur at the double bond next to the oxygen of the lactone ring in compound **8,** resulting in a covalent ether bond, as in syringolin A. More structure-activity relationship studies should be conducted to explore natural or synthetic analogues in order to develop new inhibitors.

## 3. Experimental Section

### 3.1. General Methods

NMR spectra were recorded in CDCl_3_ and Acetone-*d*_6_, and chemical shifts were referenced relative to the corresponding signals (δ_H_ 7.24/ δ_C_ 77.2 for CDCl_3_; δ_H_ 2.05/ δ_C_ 206.7 and 29.9 for Acetone-*d*_6_) measured using a Varian VNS 600 spectrometer (^1^H: 600 MHz, ^13^C: 150 MHz) or a JEOL ECA-500 (^1^H: 500 MHz, ^13^C: 125 MHz). The chemical shift values (δ) and coupling constants (*J*) are indicated in parts per million (ppm) and Hertz (Hz), respectively. All HPLC separations were carried out on an Agilent series 1260 HPLC system equipped with diode array detector. Open column chromatography was performed on silica gel 60 (70–230 mesh, Merck, Darmstadt, Germany), LiChroprep RP-18 gel (40–63 μm, Merck), and Sephadex LH-20 gel (GE Healthcare, Danderyd, Sweden). TLC was performed on silica gel 60 F_254_ (Merck), and the results were visualized with a UV detector (Vilber Lourmat, Marne La Vallée, France) and color reaction of 20% aqueous H_2_SO_4_. All solvents used for extraction and isolation were of analytical grade.

### 3.2. Cultures

The fungal strain JS900 was isolated from a rhizome of *Phragmites communis* collected from a swamp in Suncheon, South Korea. Fungal strains were identified by sequencing ITS regions with ITS1 and ITS4 primers. After a homology search against NCBI nt DB with a BlastN algorithm and phylogenetic analysis with ITS sequences from NCBI, JS900 was identified as *Pestalotiopsis sydowiana*. Fungus JS900 in 50% aqueous glycerol solution was stored in a ‒70 °C freezer at the College of Pharmacy, Duksung Women’s University (Seoul, Korea). The fungal strain was cultivated at 28 °C for 28 days in four 500 mL Erlenmeyer flasks each containing 80 g of rice and 120 mL of water.

### 3.3. Extraction and Isolation

The solid cultures were extracted with EtOAc to produce 12.3 g of extract, which was subjected to a silica gel vacuum liquid chromatography column by elution with a stepwise gradient of EtOAc in *n*-hexane (*v*/*v*), to give 14 fractions. Fraction 9 was subjected to Sephadex LH-20 chromatography (10 mm × 400 mm) and then further purified by reverse-phase HPLC (40%–100% MeOH in H_2_O over 40 min; Phenomenex Luna 5 μ C18 column (250 mm × 10.0 mm); flow rate of 2 mL/min) to give compounds **1** (t_R_ 9.0 min; 2.4 mg) and **8** (t_R_ 9.0 min; 0.8 mg). Fractions 5 and 6 were combined and rechromatographed by Sephadex LH-20 column chromatography by elution with CH_2_Cl_2_/MeOH (1/1) to yield compounds **5** (1.0 mg) and **9** (1.8 mg). Fractions 9 and 10 were rechromatographed by Sephadex LH-20 to give 15 subfractions, and recrystallization of subfractions 5–7 gave compound **9**. Subfractions 5–9 were purified by reverse phase HPLC (10%–100% MeOH in H_2_O over 45 min; Phenomenex Luna 5 μ C18 column (250 mm × 10.0 mm); flow rate of 2 mL/min) to obtain compounds **6** (t_R_ 22.8 min; 3.0 mg) and **10** (t_R_ 25.2 min; 3.1 mg). Fractions 12 and 13 were combined and further separated by Sephadex LH-20 by elution with CH_2_Cl_2_/MeOH (1/1) to produce 15 subfractions. Subfractions 12–14 were further purified by reverse phase HPLC (40%–100% MeOH in H_2_O over 45 min; Phenomenex Luna 5 μ C18 column (250 mm × 10.0 mm); flow rate of 2 mL/min) to produce compounds **2** (t_R_ 28.6 min; 3.0 mg) and **3** (t_R_ 30.2 min; 3.1 mg). Subfraction 15 was subjected to reverse phase HPLC (20%–100% MeOH in H_2_O over 45 min; Phenomenex Luna 5 μ C18 column (250 mm × 10.0 mm); flow rate of 2 mL/min) to obtain compounds **4** (t_R_ 28.5 min; 2.5 mg) and **7** (t_R_ 15.3 min; 4.2 mg).

Compound **1**: C_22_H_24_O_6_, white powder, ^1^H-NMR (CDCl_3_, 600 MHz) δ: 1.37 (s, 6H), 2.23 (3H, s), 3.20 (3H, s), 3.91(3H, s), 5.05 (2H, s), 6.20 (1H, d, *J* = 16 Hz), 6.70, (1H, d, *J* = 16 Hz), 6.36 (1H, s), 6.84 (1H, s), 6.87 (1H, d, *J* = 8.4 Hz), 7.58 (1H, d, *J* = 8.4 Hz); ^13^C-NMR (CDCl_3_, 150 MHz): 168.0, 155.4, 151.8, 147.6, 144.4, 141.5, 135.3, 132.4, 132.3, 125.9, 120.9, 119.4, 118.0, 117.8, 114.4, 78.7, 69.2, 62.9, 60.6, 21.0, 18.6; (+)ESI-MS *m/z* 385.4 [M + H]^+^.

Compound **2**: C_21_H_22_O_7_, white powder, [α]_D_ = +21.5 (*c* 0.1, MeOH); ^1^H-NMR (CDCl_3_, 600 MHz) δ: 7.54 (1H, d, *J* = 8.4 Hz), 6.84 (1H, s), 6.22 (1H, d, *J* = 5.4 Hz), 6.33 (1H, s), 5.00 (2H, s), 4.98 (1H, d, *J* = 4.8 Hz), 4.85 (2H, s), 4.15 (1H, d, *J* = 4.8 Hz), 3.93 (3H, s), 2.21 (3H, s), 1.76 (3H, s); ^13^C-NMR (CDCl_3_, 150 MHz): 167.8, 154.8, 151.8, 147.5, 143.9, 141.3, 135.1, 132.6, 132.0, 125.7, 120.7, 118.9, 117.8, 117.3, 113.0, 78.2, 69.1, 62.2, 20.8, 18.7; (+)ESI-MS *m/z* 387.4 [M + H]^+^.

Compound **3**: C_21_H_22_O_7_, white powder, [α]_D_ = −15.8 (*c* 0.1, MeOH); ^1^H-NMR (CDCl_3_, 600 MHz) δ: 7.56 (1H, d, *J* = 8.7 Hz), 6,83 (2H, d, *J* = 4.5 Hz), 6.32 (1H, s), 5.04 (1H, d, *J* = 14 Hz), 5.03 (1H, s), 4.85 (1H, d, *J* = 14 Hz), 4.28 (1H, d, *J* = 5.7 Hz), 3.94 (3H, s), 2.21 (3H, s), 1.70 (3H, s); ^13^C-NMR (CDCl_3_, 150 MHz): 168.0, 155.4, 151.8, 147.6, 144.4, 141.5, 135.3, 132.4, 132.3, 125.9, 120.9, 119.4, 118.0, 117.8, 114.4, 78.7, 69.2, 62.9, 60.6, 21.0, 18.6; (+)ESI-MS *m/z* 387.4 [M + H]^+^.

Compound **4**: C_21_H_22_O_6_, white powder, ^1^H-NMR (CDCl_3_, 600 MHz) δ: 1.30 (6H, s), 2.23 (3H, s), 3.9 (3H, s), 5.05 (2H, s), 6,85 (1H, d, *J* = 8.4 Hz), 6.82 (1H, s), 6.39 (1H, s), 5.98 (1H, s), 7.55 (1H, d, *J* = 8.7 Hz); ^13^C-NMR (CDCl_3_, 150 MHz): 169.5, 156.2, 153.2, 149.7, 143.2, 141.8, 137.0, 132.1, 130.9, 128.1, 122.3, 121.3, 120.3, 119.5, 119.3, 70.5, 63.3, 29.9 (2 × CH_3_), 20.8; (+)ESI-MS *m/z* 371.2 [M + H]^+^.

Compound **5**: C_7_H_10_O_4_, white powder, [α]_D_ = +10.2 (*c* 0.1, MeOH); ^1^H-NMR (CDCl_3_, 300 MHz) δ: 5.13 (1H, d, *J* = 1.5 Hz), 4.48 (1H, dq, *J* = 3.9, 12 Hz), 3.90 (1H, br d, *J* = 12 Hz), 3.75 (3H, s), 2.77 (1H, ddd, *J* = 1.8, 12, 17 Hz), 2.25 (1H, dd, *J* = 3.9, 17 Hz), 2.03 (1H, m); ^13^C-NMR (CDCl_3_, 75 MHz): 172.7, 166.5, 89.8, 76.0, 63.5, 56.0, 28.6; (+)ESI-MS *m/z* 159.2 [M + H]^+^.

Compound **6**: C_10_H_14_O_5_, white powder, [α]_D_ = −5.8 (*c* 0.1, MeOH); ^1^H-NMR (Acetone-*d*_6_, 600 MHz) δ: 0.84 (3H, d, *J* = 7.2 Hz), 1.46 (3H, s), 3.90 (3H, s), 3.94 (1H, m), 5.40 (1H, d, *J* = 2.4 Hz), 6.21 (1H, d, *J* = 2.4 Hz); ^13^C-NMR (CDCl_3_, 150 MHz): 172.3, 169.9, 98.9, 87.9, 76.1, 71.9, 56.6, 23.7, 18.0; (+)ESI-MS *m/z* 237.0 [M + Na]^+^.

Compound **7**: C_10_H_14_O_4_, white powder, [α]_D_ = +11.2 (*c* 0.1, MeOH); ^1^H-NMR (CDCl_3_, 600 MHz) δ: 1.19 (3H, d, *J* = 6.3 Hz), 1.23 (3H, d, *J* = 6.9 Hz), 2.53 (1H, m), 4.01 (1H, m), 5.40 (1H, d, *J* = 2.1 Hz), 5.83 (1H, d, *J* = 2.1 Hz); (+)ESI-MS *m/z* 221.0 [M + Na]^+^.

Compound **8**: C_10_H_12_O_3_, white powder, ^1^H-NMR (CDCl_3_, 600 MHz) δ: 1.71 (3H, d, *J* = 6.3 Hz), 1.80 (3H, s), 3.80 (3H, s), 5.42 (1H, d, *J* = 2.7 Hz), 5.90 (1H, d, *J* = 2.7 Hz), 6.73 (1H, q, *J* = 6.3 Hz); (+)ESI-MS *m/z* 202.9 [M + Na]^+^.

Compound **9**: C_11_H_18_O_5_, white powder, [α]_D_ = −32.0 (*c* 0.1, MeOH); ^1^H-NMR (Acetone-*d*_6_, 600 MHz) δ: 0.92 (3H, t, *J* = 6.6 Hz), 1.21 (1H, m), 1.33–1.67 (3H, m), 2.32 (1H, dt, *J* = 3.6, 13 Hz), 2.87 (1H, dt, *J* = 3.0, 4.2 Hz), 3.48 (1H, t, *J* = 3.6 Hz), 3.75 (1H, m), 3.80 (3H, s), 4.50 ( 1H, dt, *J* = 3.9, 17.4 Hz), 5.11 (1H, s); ^13^C-NMR (CDCl_3_, 150 MHz): 173.4, 166.6, 89.7, 78.1, 73.8, 70.9, 56.2, 36.0, 29.3, 18.8, 13.9; (+)ESI-MS *m/z* 231.2 [M + H]^+^.

Compound **10**: C_11_H_18_O_5_, white powder, [α]_D_ = −15.0 (*c* 0.1, MeOH); ^1^H-NMR (Acetone-*d*_6_, 600 MHz) δ: 0.90 (3H, d, *J* = 7.5 Hz), 1.34–1.45 (2H, m), 1,46–1.59 (1H, m), 1.80–1.84 (1H, m), 2.25 (1H, dd, *J* = 3.9, 17 Hz), 2.94 (1H, ddd, *J* = 1.3, 13, 17 Hz), 3.72 (1H, m), 3.78 (3H, s), 3.88 (1H, m), 4.75 (1H, dt, *J* = 1.8, 13 Hz), 5.08 (1H, d, *J* = 1.3 Hz); ^13^C-NMR (Acetone-*d*_6_, 150 MHz): 174.7, 167.1, 90.4, 75.9, 75.7, 70.4, 56.7, 37.1, 19.3, 14.6; (+)ESI-MS *m/z* 253.1 [M + Na]^+^.

### 3.4. Proteasome Inhibition Assay

The inhibition assay was performed as follows [9]. A commercially available 20S proteasome assay kit was employed (Enzo^®^ Life Sciences, BML-AK740). A 0.03% sodium dodecyl sulfate was added to the assay buffer in order to activate the chymotrypsin-like activity of the 20S proteasome. The enriched proteasome fraction was prepared to an assay concentration of 50 μg/mL using the assay buffer. The isolated compounds were dissolved in DMSO to a concentration of 4 mg/mL for screening. The activated assay buffer, inhibitor (samples and positive control) solutions, and enzyme were added to each well of a plate, and the plate was preincubated for 10 min at 37 °C to allow for interaction between the inhibitor and enzyme. The final concentrations of enzyme, the samples, and epoxomicin were 10 μg/mL, 0.1 mg/mL, and 0.5 μM, respectively, for the screening assay. The enzymatic reaction was initiated by the addition of SUC-LLVY-AMC substrate at a concentration of 10 μM. Chymotrypsin-like enzymatic activity of the proteasome was determined by measurement of the generation of free AMC on a fluorescence plate reader (FluorOptima, BMG LabTech Ltd., Aylesbury, UK) capable of excitation at a wavelength of 355 nm and detection of emitted light at 460 nm. Epoxomicin, a potent and irreversible inhibitor of 20S proteasome chymotrypsin-like activity, was used as the positive control (IC_50_ = 72 nM). This compound can also inhibit trypsin-like and peptidyl-glutamyl peptide hydrolase activities of proteasome, but at a 100- and 1000-fold slower rate, respectively. For compounds **1**–**3**, **5**, and **8**–**10** with potent inhibitory activities in the initial screening assay, a dilution series of each inhibitor was performed to determine IC_50_ values.

## Figures and Tables

**Table 1:** 20S proteasome inhibitory activities of compounds **1**–**10**.

Compound	IC_50_^a^ (μM)	Compound	IC_50_^a^ (μM)
**1**	30.5 ± 1.5	7	>100
**2**	18.5 ± 4.2	8	1.2 ± 0.3
**3**	12.4 ± 1.1	9	8.9 ± 1.5
**4**	>100	10	7.9 ± 1.8
**5**	20.5 ± 2.3	Epoxomicin ^b^	0.072
**6**	>100		

^a^ IC_50_ values were determined by regression analyses and expressed as mean ± standard deviation of three replicates. ^b^ Epoxomicin was used as a positive control.
